# Isolation and Characterization of Milk Exosomes for Use in Advanced Therapies

**DOI:** 10.3390/biom14070810

**Published:** 2024-07-08

**Authors:** Ana Medel-Martinez, Ana Redrado-Osta, Alejandra Crespo-Barreda, Maria Sancho-Albero, Lourdes Sánchez, Víctor Sebastián, María Pardo, Antonio de la Vieja, Pilar Martín-Duque

**Affiliations:** 1 Instituto de Investigaciones Sanitarias de Aragón (IIS Aragón), 50009 Zaragoza, Spainmsancho@unizar.es (M.S.-A.); victorse@unizar.es (V.S.); 2 Instituto Aragonés de Ciencias de la Salud (IACS), 50009 Zaragoza, Spain; 3 Instituto de Nanociencia y Materiales de Aragon (INMA), CSIC-Universidad de Zaragoza, Campus Rio Ebro, Edificio I+D, C/Poeta Mariano Esquillor, s/n, 50018 Zaragoza, Spain; 4 Networking Research Center in Biomaterials, Bioengineering and Nanomedicine (CIBERBBN), Instituto de Salud Carlos III, 28029 Madrid, Spain; 5 Department of Chemical and Environmental Engineering, University of Zaragoza, Campus Rio Ebro, C/María de Luna, 3, 50018 Zaragoza, Spain; 6 Instituto Agroalimentario de Aragón, University of Zaragoza, 50018 Zaragoza, Spain; lsanpan@unizar.es; 7 Grupo Obesidómica, Área de Endocrinología, Instituto de Investigación Sanitaria de Santiago de Compostela (IDIS), CIBEROBN (ISCIII), Xerencia de Xestión Integrada de Santiago (XXIS/SERGAS), 15706 Santiago de Compostela, Spain; 8 Endocrine Tumors Unit, Chronic Disease Program (UFIEC), Instituto de Salud Carlos III, 28222 Majadahonda, Spain; adelavieja@isciii.es; 9 Nanomedicines and Nanotherapies Unit, Departament of Development of Medicines and Advanced Therapies, Instituto de Salud Carlos III, 28222 Majadahonda, Spain; 10 Surgery Department, Medical School, University of Zaragoza, 50009 Zaragoza, Spain

**Keywords:** milk exosomes, extraction protocol, casein, milk fat globules, rennet, storage, characterization

## Abstract

Exosomes are cell-derived extracellular vesicles (EVs) with diameters between 30 and 120 nm. In recent years, several studies have evaluated the therapeutic potential of exosomes derived from different fluids due to their low immunogenicity and high biocompatibility. However, producing exosomes on a large scale is still challenging. One of the fluids from which they could be isolated in large quantities is milk. Moreover, regeneration is a well-known property of milk. The present work seeks to optimize a method for isolating exosomes from bovine and human milk, comparing different storage conditions and different extraction protocols. We found differences in the yield extraction associated with pre-storage milk conditions and observed some differences according to the processing agent. When we removed milk fat globules and added rennet before freezing, we obtained a cleaner final fraction. In summary, we attempted to optimize a rennet-based new milk–exosome isolation method and concluded that pre-treatment, followed by freezing of samples, yielded the best exosome population.

## 1. Introduction

Exosomes are extracellular vesicles (EVs) generated by a process of invagination of the endosomal membrane, and that are released under physiological conditions, and at increased rates in pathological circumstances, including inflammatory diseases such as diabetes, cancer, and cardiovascular and neurodegenerative diseases [1]. For this reason, study of these vesicles has made a significant contribution to the understanding of several different pathologies. In fact, their function has been controversial, as they seem to play different roles under various pathological stages, depending on the cell source. On the one hand, several studies have reported exosome subpopulations as being implicated in the progression of pathologies such as cancer or neurodegenerative diseases [2,3]. On the other hand, the number of studies describing the therapeutic potential of exosomes has increased exponentially. In this sense, it has been shown that exosomes are capable of suppressing the damaging immune response in inflamed tissues, and promoting the survival and regeneration of damaged cells [4,5]. Therefore, the search for easily obtainable therapeutic exosomes constitutes an important issue.

One of the biological fluids from which exosomes can be obtained in large quantities is breast-milk, the regenerative properties of which have been known for years [6,7,8]. Apart from that, milk exosomes show other properties, such as tolerance between species, with no observed immune or inflammatory response, which makes them ideal for therapeutic use [9]. Thus, milk is an easily obtained and safe source for exosome isolation. Currently available protocols used to isolate the exosomes present in milk are limited by the presence of other contaminants which co-precipitate with exosomes. Among these non-desired components, fat and casein are some of the most abundant ones. The first one can fuse with exosomes, altering their physical properties and, therefore, their functionality. Breast-milk is a complex biological fluid, the fat content of which varies throughout the day and depending on the emptying of the breast. The fat in breast-milk is essential for the baby’s development, providing energy, supporting brain development, aiding in the absorption of vitamins, promoting growth, strengthening the immune system, and promoting digestive health.

Fat in maternal milk is similar to chylomicrons and serum lipoproteins, in that all contain triglycerides, phospholipids, cholesterol, and fat-soluble vitamins, and these compositions can be influenced by the maternal diet. All use lipoprotein particles to transport lipids, but their origins and functions differ. Chylomicrons are formed in the intestines to transport dietary fats, while various lipoproteins (VLDL, LDL, and HDL) are synthesized in the liver for lipid metabolism. In contrast, milk fat globules are synthesized in the mammary gland, and designed to provide nutrition, energy, and immune support to infants. Milk fat also contains bioactive molecules like enzymes and antibodies, which are not found in serum lipoproteins, and it is encapsulated in a complex membrane structure specific to breast-milk.

Casein is a phosphoprotein that represents between 40–80% of the total protein content of the milk, and which masks and reduces the purity of the exosomes, affecting the quantification of these EVs [10].

Ultracentrifugation is the most widely used exosome isolation strategy because it is an easy technique, with the additional advantage of not requiring sample pretreatment. However, as mentioned above, when using this approach for purifying milk-derived EVs, the exosome fraction contains large quantities of casein, making this approach less useful. For this reason, other studies have combined ultracentrifugation with other methods such as exclusion chromatography or hydrochloride-based isoelectric precipitation [9]. Although promising, these studies cannot discriminate these vesicles from other EVs or lead to exosome degradation. Therefore, optimization of an isolation method for milk exosomes has not been yet achieved.

Apart from that, pre-isolation milk storage is a major issue. Previous studies have demonstrated that characteristics and functions of exosomes can be determined by storage conditions. In this sense, Leiferman et al. evidenced a significant decrease in the size and number of exosomes due to different storage conditions [11].

Herein we have aimed to develop a complete isolation method, reproducible and easily implementable. Therefore, the first goal was to find a useful strategy to successfully eliminate casein during the isolation protocol without exosome alteration. We tested two different treatments in parallel to eliminate casein during the isolation protocol. First, a hydrochloride acid treatment which was expected to remove other precipitates was tested. Afterwards, an enzymatic treatment using chymosin was evaluated. Chymosin constitutes the principal enzyme content in rennet. This enzyme is an aspartic protease, one which can hydrolyze a specific peptide bond (Phe105-Met106) present in κ-casein, causing the aggregation of casein micelles and forming an easily removable gel. We did not observe significant differences according to the processing agent used. However, rennet was finally chosen as the most promising approach to use for discarding catalase from the isolated exosomes as we observed that hydrochloride acid decreased the exosome number and could affect exosome functionality in future applications.

Once we had selected rennet over the hydrochloride acid treatment, we focused on exploring different protocols that might combine to make the best way to retire fat globules, yielding a cleaner exosome population. As fat is more prone to breakage at lower temperatures than are other tissues [12], we guessed that a prior freezing process could eliminate these globules. We achieved this goal, designing several strategies that combined different steps for eliminating fat and freezing.

Thus, the second objective of this study was the characterization of various milk-processing approaches in order to determine exosome purity and integrity for later use. We found differences in the extraction performance according to pretreatment and the conditions associated with the freezing of the milk, concluding that discarding fat followed by freezing yielded the purest isolated EVs. In fact, our results indicated that fresh milk-derived exosomes were co-sedimented with other non-desired residues that contaminated the sample, confirming that freezing milk is optimal for exosome isolation.

We believe that milk exosomes could be great delivery agents for drug transfer to neoplasias or other pathologies. A pure population of exosomes or controlled conditions for their purification are highly desired, and this knowledge could subsequently be transferred to the clinic. As access to human milk is limited, the use of milk from other sources would be ideal. Unlimited supply and harmless access to samples would be great advantages for use in a clinic.

In summary, a new rennet-based milk-exosome-extraction approach and purification methodology have been successfully developed to overcome the aforementioned challenges. Furthermore, the possibility of freezing samples before EV isolation has been exploited to facilitate its possible implementation, opening the door to milk exosome therapeutical applications.

## 2. Materials and Methods

### 2.1. Milk Collection

Bovine milk samples (25 mL for each designed protocol) were extracted from healthy cows from the Torreconde farm (Zaragoza, Spain). Human-milk samples (25 mL for each designed protocol) were provided by donors. The project was authorized by the local ethics committee (C.P.-C.I. PI21/000 from 13 January 2021. Samples were collected, placed in sterile collection bags, and stored at 4 °C during transport. After arrival, samples were stored at −80 °C until use or directly processed, depending on the chosen strategy.

### 2.2. Milk Exosome Isolation

Three different approaches were used to explore whether removal of cream fat globules and casein proteins prior to long-term storage at −80 °C may influence exosome isolation and characterization efficiency. Also, the influence of the form of post-freeze processing on the quality and purity of the isolated EVs was explored (see graphical abstract). The tested methods were as follows: For Method A, unprocessed, whole milk was processed immediately upon arrival. For Method B, unprocessed milk was immediately stored at −80 °C for more than a week and processed immediately after thawing. Method C required the degreasing of the sample, the addition of rennet, and storage at −80 °C for more than a week before processing.

#### 2.2.1. Degreasing

Samples were subjected to two rounds of centrifugation at 3000× *g* for 10 min at 4 °C to remove the cream layer containing fat globules and the resulting pellet.

#### 2.2.2. Acid Treatment

Samples were warmed at 37 °C and hydrochloric acid (HCl) 1 M was added until the pH of the solution was in the range of 4.6–4.8. Subsequently, samples were centrifuged at 5000× *g* and 4 °C for 30 min to remove casein traces and other protein contaminants.

#### 2.2.3. Enzymatic Treatment

For the use of the coagulant Qualact (Altecsa S.A., Ciudad de México, CDMX, Mexico), samples were treated by adding different concentrations ranging from 0% to 5% (*vol*/*vol*) for protocol development, and then incubated at 37 °C for 20 min. After setting up the protocol, a concentration of 0.5% (*vol*/*vol*) was established. While using rennin derived from Mucor Miehei and calf stomach (Sigma, St. Louis, MO, USA), we followed the specific instructions provided with the products (~0.1 U/μγ protein for rennin and ≥20 units/mg protein for calf stomach). The samples were incubated with rennin and calf stomach at 37 °C and 30 °C, respectively. After treatment, the solutions were centrifuged at 5000× *g* for 30 min at 4 °C to discard casein residues and other cellular proteins.

#### 2.2.4. Exosome Isolation

After treatment, samples were centrifuged at 12,000× *g* at 4 °C for 20 min to eliminate cell debris, other microvesicles, and residual creams. Then, supernatant was centrifuged again at 100,000× *g* at 4 °C for 70 min to precipitate exosomes. The exosome pellet was washed with PBS and centrifuged again at 100,000× *g* at 4 °C for 70 min. Finally, exosomes were resuspended in 500 μL of PBS and aliquoted into several vials which were stored frozen at −80 °C until used.

### 2.3. Isolation of Milk Fat Globules

The milk fat globule fraction was obtained from raw milk that was heated at 50 °C and separated into cream and skim milk using a cream separator (ARR-DES 125, Suministros Químicos Arroyo, Santander, Spain). Cream was washed 3 times with Milli-Q water (Millipore, Billerica, MA, USA) to remove caseins and whey proteins and centrifuged between each wash at 3400× *g* for 15 min at 4 °C. Then, the washed cream was churned to obtain butter and buttermilk. Butter was heated at 40 °C for 15 min and centrifuged at 3000× *g* for 15 min, resulting in an upper phase (oil) and a lower phase (butter serum). Buttermilk and butter serum were mixed in a proportion of 7:1 (*vol*/*vol*), filtered through glass wool, and acidified to pH 4.8 with 1 M HCl. The mixture was stirred for 30 min and centrifuged at 40,000× *g* for 30 min at 4 °C to obtain the precipitated milk fat globule fraction. Total protein content was determined by the bicinchoninic acid (BCA) protein assay kit (Pierce Biotechnology, Rockford, IL, USA).

### 2.4. Transmission Electron Microscopy

Exosome size and morphology were evaluated by transmission electron microscopy (TEM) using a T20-FEI Tecnai thermoionic microscope (Thermo Fisher Scientific, Waltham, MA, USA) at the Advanced Microscopy Laboratory (LMA)-ELECMI ICTS. Samples were fixed on formvar-coated carbon grids and stained with 5µL phosphotungstenic acid (3% in dH_2_O). Subsequently, the grids were examined under the microscope, operated at 200 kV with a LaB6 electron source fitted with a “SupperTwin^®^” objective lens allowing a point-to-point resolution of 2.4.

### 2.5. Nanoparticle Tracking Analysis (NTA)

The hydrodynamic diameter and the concentration of the exosomes were characterized by NTA. Exosome fractions were diluted 250/1000-fold in 1 mL PBS to reach an optimal particle concentration. Then, vesicle suspensions were analyzed using a Nanosight NS300 (NanoSight Ltd., Amesbury, UK) equipped with a 405 nm blue laser to estimate the size and concentration of the isolated particles. Samples were infused at rate of 50 (arbitrary units) by using a syringe pump and the flow-cell top-plate. A total of 5 videos of 60 s duration were taken with a frame rate of 25 frames/s, and particle movement was captured at a fixed detection threshold of 9 and analyzed using NTA software (version NTA 3.4 Build 3.4.003).

### 2.6. Protein Quantification

To quantify the total protein content of the isolated exosomes, the bicinchoninic acid (BCA) method (Thermo Fischer Scientific, Waltham, MA, USA) was employed. Firstly, 12.5 μL of commercial standard albumin (0–2000 μL/mL) and 12.5 μL of each sample were added to the wells of a 96-well plate. Then, 100 μL of the BCA-containing solution was added and samples were incubated for 30 min at 37 °C. Absorbance was measured at 562 nm using a Synergy HT plate reader (BioTek, Winooski, VT, USA). Finally, the protein concentration was calculated by extrapolating the absorbance value of the samples against the absorbance value of the standards of known concentration.

### 2.7. Western Blot

Western blot (WB) analysis was performed to determine the specific exosome surface proteins and casein expression. Briefly, the isolated exosome pellet was resuspended in 100 μL of RIPA buffer (Sigma, USA) for Western blot studies and quantified by BCA, as previously described. Then, 100 μg of exosomes were suspended in Laemmli buffer 1× (Sigma Aldrich, USA) and boiled at 95 °C for a duration of 5 min. Proteins were separated in a 10% SDS-PAGE gel at 30 mV. Subsequently, they were transferred by wet electro-transfer onto nitrocellulose membranes. Blots were blocked for 1 h in 5% BSA in TBS with 0.5% Tween-20. Then, primary antibodies (anti-CD9, 1:250 (Biorad, Hercules, CA, USA); anti-CD81, 1:250 (Santa Cruz Biotechnology, Dallas, TX, USA); anti-ALIX, 1:250 (Cell Signaling Technology, Danvers, MA, USA); anti-TSG101, 1:1000 (Santa Cruz Biotechnology, USA); anti-CD63, 1:250 (Santa Cruz Biotechnology, USA); and anti-casein, 1:100 (Abcam, Cambridge, UK)) were added for overnight incubation at 4 °C. After triplicate washes with TBS-Tween, secondary antibody conjugated with HRP was added for 1 h at room temperature. The blot was finally developed with Supreme ECL HRP Substrate (NzyTech, Lisboa, Portugal) and imaged with ChemiDoc XRS (Bio-Rad, Hercules, CA, USA).

### 2.8. Exosome Antibody Array

Identification of the exosome biomarkers of human breast-milk was performed using the Exo-Check Exosome Ab Array (System Biosciences, Palo Alto, CA, USA) kit by following the manufacturer’s instructions. Briefly, samples (50 µg) were lysed, labelled, and then incubated at room temperature for 30 min. Then, any excess of labeling reagent was removed using the provided columns. Subsequently, the eluted labeled exosome lysate was blocked and then incubated in the antibody array capture membrane overnight at 4 °C. The membrane was washed, incubated for 30 min at RT with detection buffer, and washed again. Finally, a blot was developed with Supreme ECL HRP Substrate (NzyTech, Lisboa, Portugal) and imaged with ChemiDoc XRS (Bio-Rad, Hercules, CA, USA).

### 2.9. Casein Analysis by ELISA

The amount of casein present in the bovine milk-derived exosomes was measured using a SENSISpec ELISA Casein kit (Immunolab, GKassel, Hessen, Germany) by following the manufacturer’s recommendations. Briefly, samples and standards were incubated for 20 min and washed several times. Then, peroxidase-conjugated anti-casein antibody was added and incubated for 20 min. Finally, the wells were washed and a substrate solution was added that reacted with peroxidase, giving a blue color, for the purposes of measurement.

The casein concentration was multiplied by the specific conversion factor of the product, which in the case of whole milk was 42. Casein concentration was obtained from a calibration curve in ppm. Absorbance was read at 450 nm using a Biotek Synergy HT microplate reader (Agilent, Santa Clara, CA, USA). 

### 2.10. SP-IRIS/Exoview Studies

To analyze the EVs, we used the new single-particle interferometric reflectance imaging sensor (SP-IRIS) ExoView^®^ R200 (now Leprechaun from Unchained labs, Pleasanton, CA, USA), which allows us to carry out EV particle size analysis and measure the EV concentration, EV phenotype, and biomarker colocalization. This technology is based on the single-particle interferometric reflectance imaging sensor technique, which has previously been used to detect viruses and EVs down to 50 nm in size. Complete characterization of the exosomes extracted by the different methods was performed using ExoViewHumanTetraspanin chips (NAVEV-TETRA-C, Nanoview Biosciences, Boston, MA, USA) designed to capture EVs with anti-DC63, anti-CD81, anti-CD9 and human IgG as isotype controls. Chips were pre-scanned following the instructions provided in the manufacturer’s protocol to generate baseline measurements of pre-adhered particles before sample incubation. For sample incubation, 50 µL of EVs (diluted at 1:25 in incubation solution buffer) were carefully loaded onto the pre-scanned chip and incubated overnight at room temperature without agitation. The incubation was carried out in sealed 12-well plates. Then, several washes were performed according to the manufacturer’s protocol before the addition, for purposes of detection, of 1 µg/mL of fluorescently labelled antibodies provided with the kit: anti-CD9 kit (CF 488A), anti-CD81 (CF 555A), and anti-CD63 (CF 647A) (Unchained labs, Pleasanton, CA, USA) The resulting mix was incubated for one hour with gentle agitation. The chips were then washed with different solutions and Milli-Q water, and then dried.

The acquired images were analyzed using ExoView Analyzer 3.1.4 software (Nanoview Biosciences; now Unchained labs). Number and size data were obtained by means of the total particle number captured by tetraspanin-scale analysis. Fluorescence intensity data were normalized by the number of particles captured by each of the antibody linkers.

## 3. Results

### 3.1. Analysis of the Extraction and Isolation Protocol for Exosomes Present in Milk

In order to reduce the residues of casein present in the isolated exosomes and to analyze their impact on the latter’s morphology and integrity, two different novel methodologies were evaluated: (1) acid treatment and (2) enzymatic treatment. Due to the difficulties involved in obtaining human-milk donors, the initial evaluation of the exosome isolation protocols was performed using bovine milk samples [13,14].

**Figure 1:**
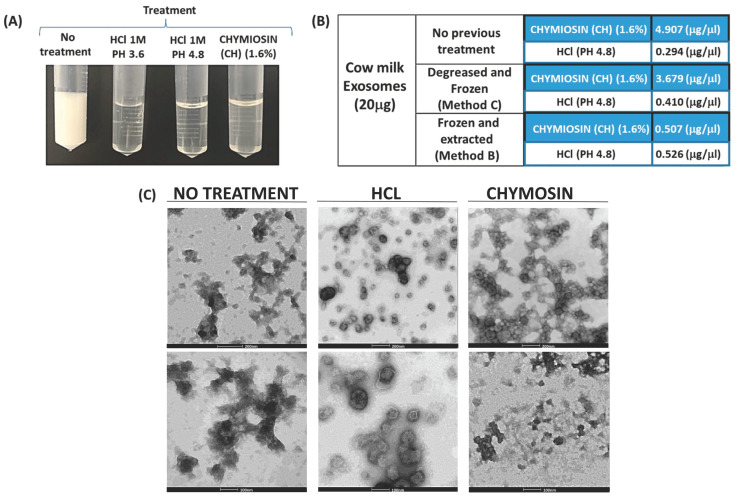
(**A**) Visual determination of bovine milk samples before isolation of the exosomes. Comparison of the pre-treatments: from left to right, non-treated sample, acid treatment (HCl at different pH), and enzymatic digestion with rennet (using Qualact (Altecsa S.A., Ciudad de México, CDMX, Mexico)). (**B**) Quantification of total protein obtained by BCA from the isolated exosomal samples to determine the exosomal content. (**C**) TEM images of the processed exosomes.

Apart from exosome morphology and integrity, we assessed the amounts of these nanovesicles by different techniques. Therefore, we first tested our treatments by visual inspection. In both treatments, it was clearly observed that the amount of casein present in the samples decreased considerably, since the coloration of the samples changed from white to transparent (Figure 1A). After inspection, both procedures seemed sufficiently useful to justify proceeding with the subsequent isolation of exosomes present in the milk.

Then, the vesicles were quantified in terms of total protein amount concentration by BCA. Protein quantification of processed samples using different protocols allowed us to approximate the exosome content in the samples previously extracted and treated with rennet or acid. Our results indicated that acid-treated samples showed a higher decrease in protein amount than the enzymatically treated preparations (Figure 1B).

We finally explored the integrity and morphology of both treated milk exosomes by TEM (Figure 1C). In general, no treated samples showed agglomerated and contaminated exosome populations, and it was difficult to find isolated exosomes (they seemed to be surrounded and coated by an organic shell of undesired residues). By contrast, treated samples showed a cleaner fraction where individual and isolated exosomes could be easily located. TEM images of acid- and enzyme-treated samples confirmed the presence of exosomes with a rounded shape and diameters of approximately 100 nm.

However, a decrease in exosome amount was observed in hydrochloric acid (HCl)-treated preparations, which could be related to the exosome lysis caused by the aggressive acid treatment. On the contrary, chymosin- or rennet-treated samples showed a cleaner appearance, without impurities, within which individual exosomes could be easily visualized, demonstrating that this treatment maintained the structure and integrity of the exosomes.

Since rennet treatment (Q) did not alter exosome morphology, showed a higher efficiency when purifying the vesicles, and did not add impurities, we decided to establish this treatment as the optimum purification protocol and use it for the subsequent experiments. However, to remove as much casein as possible, the amount of rennet incubated with the samples was optimized.

**Figure 2:**
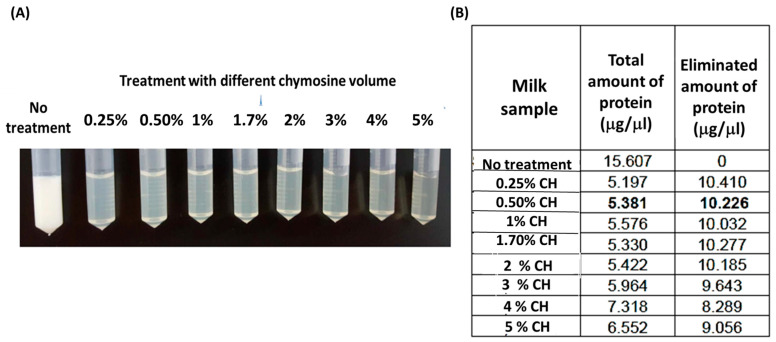
(**A**) Visual determination of bovine milk samples treated with different percentages of chymosine (expressed in *vol*/*vol*). (**B**) Quantification by BCA of the protein content eliminated after the different chymosine treatments, which is possibly related to the amount of casein precipitated and eliminated.

For this purpose, we added different volumes of the coagulant chymosin (CH), ranging between 0.25% and 5%, in a *vol*/*vol* (Figure 2A). Our results showed that, despite gradually increasing the volume of rennet added, the eliminated protein amount (by coagulation and precipitation) was similar in all the samples (Figure 2B). Therefore, a volume of 0.5% (*v*/*v*) was established as the optimal condition for the removal of casein and other proteins.

### 3.2. Analysis of Freezing and Processing Protocols

The next aspect we aimed to evaluate was the influence of the freezing of the milk, prior to the EV isolation, on the quality of the purified exosomes. Pre-freezing samples before EV isolation would be a promising advantage due to the difficulty involved in collecting and acquiring samples, something which would be particularly useful in the affected industries.

**Scheme 1:**
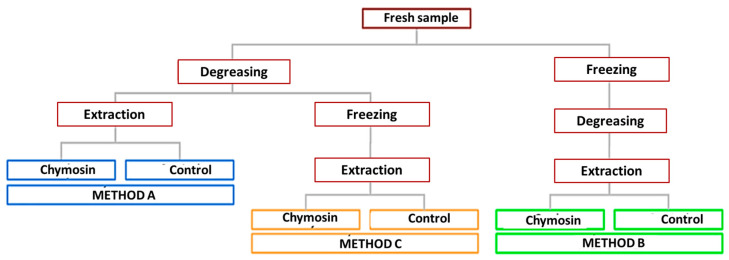
Summary of the different processing steps used in the 3 different methods. Method A began with a fresh sample and the result was extracted directly from the isolate. Method B is from a frozen sample and the result was extracted after the de-freezing process. In Method C, samples underwent a degreasing process before the sample was frozen.

Furthermore, as mentioned above, freezing proved to be an easy way to remove fat. In a previous work, it has been observed that directly pre-freezing milk leads to a non-desired co-sedimentation of apoptotic vesicles together with the exosome fraction. For this reason, Zonneveld et al. proposed, in 2014, removing milk fat globules and cells before freezing samples [15]. Based on these results, several milk pre-processing strategies, performed before EV isolation, were tested in this investigation. In particular, defatting and freezing protocols were combined with the enzymatic treatment optimized in this study, as shown below in Scheme 1.

Considering the above, human breast-milk and bovine milk seem to be promising in translational medicine; both were used as exosome sources. However, the differences in composition between human breast-milk and bovine milk were considered to optimize the processing protocol based on the milk source. In particular, human milk contains a higher amount of lipids and carbohydrates (such as lactose) and a lower protein concentration (mainly casein).

#### 3.2.1. Bovine Milk Processing

As the enzymatic treatment protocol was already developed and optimized in bovine milk, the degrees of influence of several processing conditions on the exosome isolated yield and on the vesicles’ morphology were assessed.

Firstly, we compared the exosome concentration obtained by the different methods (in terms of total protein amounts obtained by BCA), and the exosome concentration (measured by NTA) was also analyzed.

**Figure 3:**
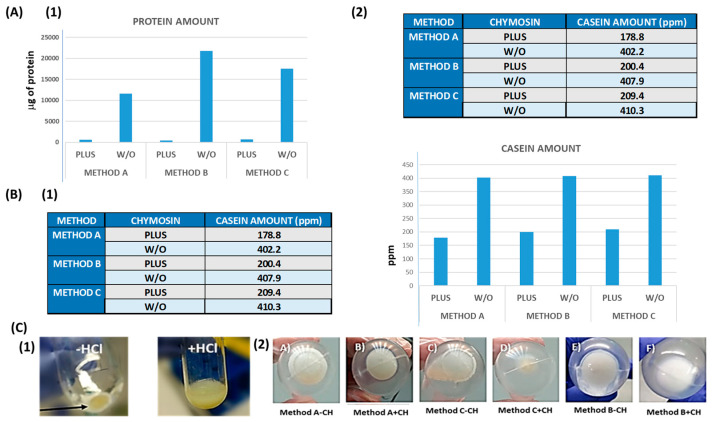
(**A**). Determination of the total protein concentration in the exosomes isolated by the different methods (*n* = 6). To calculate the values, the BCA method is used, and the absorbance of each sample is obtained. A marked decrease in the total protein amount of the treated samples was observed. (**B**). Analysis of the amounts of casein extracted from the samples by the different approaches. These values are determined by an ELISA test. There is a decrease in the casein amount in the rennet-treated samples. However, the decrease is not as high as in the total protein amount, suggesting that other proteins are also eliminated. (**C**). Observational study of the precipitated protein according to the methodologies studied by (1) precipitation with acids or (2) enzymatic precipitation. Differences could be observed on the pellet size according to the methodology for the extraction.

Figure 3A shows the protein amount (quantified by BCA) present in the processed milk, as determined by the different methodologies. As expected, when rennet was added to samples, the amount of total protein was much lower compared to non-treated samples. That fact made us think that the decrease could be due to the elimination of a large part of the casein.

For that reason, the next objective was to analyze the amount of casein present in samples by use of ELISA (Figure 3B). Predictably, our results showed a marked decrease in the casein amounts in the rennet-treated samples, but it was not as marked in the total protein quantification results. Although, in milk, caseins are part of 80% of the bovine milk proteins, the other 20% comprise many other whey proteins (lactoglobulins, lactalbumins, immunoglobulins, etc.), so it is possible that there was a large elimination of other dairy proteins in the samples. In our case, that elimination is an advantage that would allow us to obtain purer samples.

**Figure 4:**
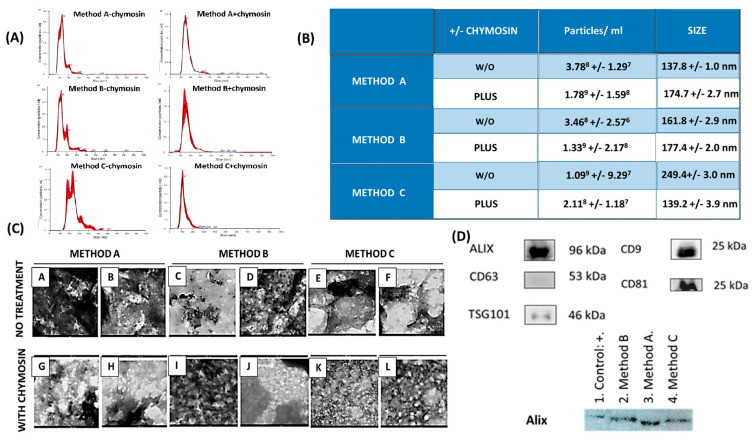
Analysis of the characterization of differences in bovine milk-derived exosomes subjected to different protocols. (**A**,**B**) Profile and size distribution measured by NTA. (**C**) TEM images representation at different magnifications. Images show the differences on clarity and the amount of inespecific organic material (**D**) Analysis of presence of exosome biomarkers by Western blot, also for milk obtained by different methods (all with enzymatic treatment).

Considering previous results, the next objective was to analyze the amounts of casein present in the samples by using ELISA (Figure 3B). Predictably, our results showed a higher amount of casein in free rennet-treated samples, indicating that a large part of the total protein detected by the BCA method is composed of casein. Treated samples presented similar amounts of casein, regardless of the processing protocol. In Figure 3C, one can observe deep differences in the levels of precipitated protein depending on the different methodologies studied. Subsequently, exosome concentrations were measured by NTA (Figure 4A). The NTA of the pooled exosomes identified the mean total yield of exosomes as 1 × 10^9^ particles/mL (treated samples) and 1 × 10^8^ particles/mL (untreated samples) (Figure 4B). When extrapolating back to the initial volume of exosome resuspension solution, no significant differences were found. Furthermore, the size distributions obtained by NTA revealed similar average-diameter sizes (Figure 4A). The NTA profiles show the average profile of the triplicate samples passed through the equipment. Although they appear to be different, they all show a homogeneous peak which contains the majority of the vesicle population. However, the dispersion shown in the untreated samples indicates that there are other populations of vesicles of different sizes, which could correspond to the contaminants in the samples which were eliminated with the treatment.

Then, electronic microscopy analysis was performed to assess if the different methods combined with the enzymatic treatment affected the stability, morphology, and dimensions of milk-derived exosomes. As shown in Figure 4C, non-treated exosome samples were characterized by the exhibition of a population in which exosomes were indistinguishable from casein. By contrast, treated samples resulted in a clear exosome fraction with a proper exosome diameter (140–170 nm, approximately), further corroborating the usefulness of the proposed enzymatic treatment. No significant differences were apparent with respect to the processing condition.

Finally, to confirm the exosomal nature of the isolated vesicles, we included a treated sample and performed a Western blot analysis of different exosome biomarkers (such as CD9, TSG, ALIX, CD81, and CD63), the signals of which are clearly observed in the different blots.

Based on these results, it was concluded that the possibility of isolating milk exosomes in frozen samples isolated from bovine milk was viable. Although interesting, the use of bovine milk presented some objections, such as tolerance between species. This fact led us to explore the possibility of using our exosome isolation protocol in human-milk samples.

#### 3.2.2. Human Breast-Milk

##### Comparison of Rennet Efficacy in Human Breast Samples

Firstly, the effect of freezing human breast-milk samples on exosome isolation quality, following the methodology used for the bovine exosome sample, was assessed. However, considering that human milk presents a different composition than bovine milk, the effectiveness of rennet in human-milk samples was evaluated.

There are several changes in the kappa-casein and beta-casein concentrations in human milk during lactation. In particular, human breast-milk presents a lower casein concentration (30%, compared to 80% in bovine milk) and a different kappa-to-beta-chain ratio [16]. Based on these characteristics, we compared the effects of three different rennet types (including the one previously used for bovine milk), using the same protocols. Qualact is a liquid rennet made from the fermentation of enzymes of microbiological origin at 700 IMCU/mL. The Mucor Miehei used here is an acid-type protease from the zygomycete mold Mucor, which is similar to rennin or calf rennet. Calf stomach rennet is the third type of rennet, and is widely used. Therefore, we compared rennets with animal, microbiological, and fungal origins.

**Figure 5:**
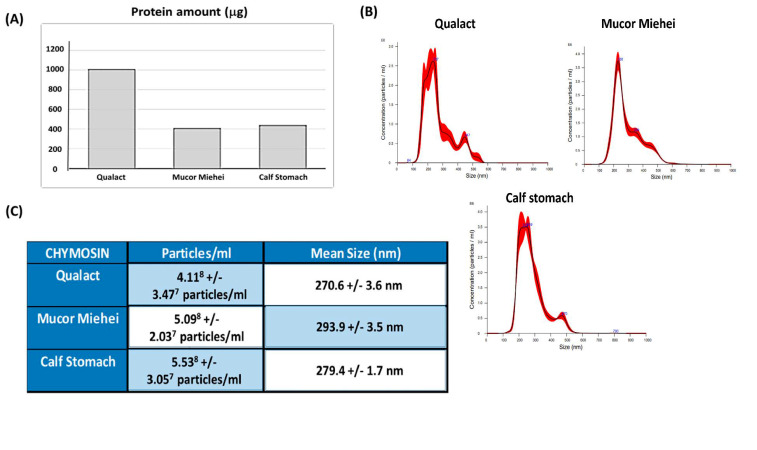
Analysis of the differences in human breast-milk-derived exosomes incubated with different rennets. (**A**) Detection of total protein amount by BCA after exosome isolation. (**B**) Size distribution, as determined by NTA. (**C**) Data as to the numbers and diameters of vesicles isolated by using the same protocol, but with rennets of different origin.

The BCA method confirmed a significant decrease in the protein amount when incubation proceeded with Mucor Miehei and calf stomach rennets, which suggested the higher efficiency of these treatments (Figure 5A).

Furthermore, analysis of exosomes isolated from different coagulation rennets by NTA confirmed that there were no differences in the amounts of exosomes obtained or the distribution between the protocols. As the numbers of exosomes obtained were similar, we could assume that the decrease in the protein obtained, as described in Figure 5A, was due to the precipitation of the casein and other milk proteins.

Only the technique used for bovine samples (Qualact) showed a population of slightly bigger size, as compared with the other treated samples (Figure 5B,C).

The bigger particles present in the Qualact-treated samples could be attributed to aggregates created due to the interactions with fat vesicles and casein. Therefore, the Mucor miehei rennet was established in the protocol for carrying out the subsequent experiments with human-milk samples

##### Comparison of Different Extraction Protocols in Human Samples

**Figure 6:**
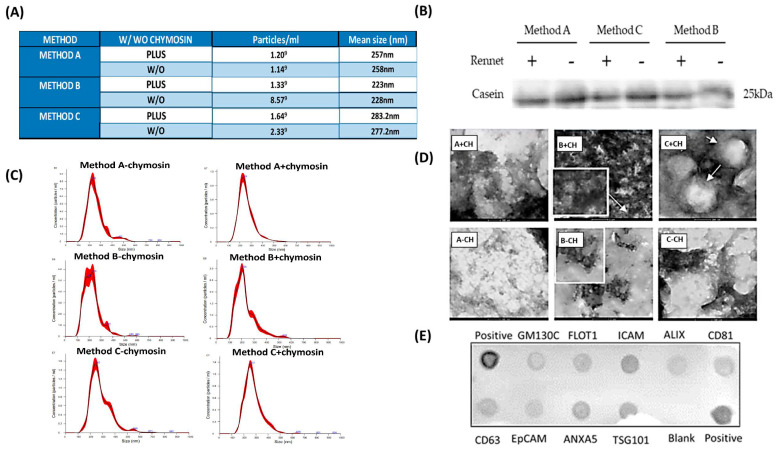
Analysis of the characterization of differences in human breast-milk-derived exosomes subjected to different conditions. (**A**) Detection of number and size, as determined by NTA. (**B**) Casein detection for the sample, extracted by Western blot. (**C**) Size distribution profile, by NTA. (**D**) Representations of TEM images of the samples extracted with the different protocols (+ or − chymosin (CH)). Scale bar: 100 nm. Arrows show isolated exosomes on the samples (**E**) Analysis of presence of exosome biomarkers by exosome antibody array using Method C + CH.

Firstly, the total values for the vesicles extracted were quantified using NTA (Figure 6A). Also, the amount of casein in each extract was evaluated by Western blot (Figure 6B). The values obtained by method “B” showed a slight decrease in the quantities of casein, compared with the other two methods.

NTA results showed a similar distribution, comparable in all protocols (Figure 6C). Vesicles with diameters of around 250 nm were observed, which indicated that the diameters of human-milk exosomes isolated with our protocol were much larger than those associated with the bovine ones.

TEM images showed significant differences, depending on the chosen method, as seen in Figure 6D. Samples derived from methods A and B exhibited an organic shell, which can probably be attributed to protein derived from non-eliminated casein. By contrast, samples obtained from Method C allowed the visualization of exosomes, due to a notable reduction in inhibitory signals and impediments to electronic passage.

We finally selected Method C as the optimum procedure, used to isolate the purest exosome population. Figure 6E includes the expression of specific exosomal markers (flotilin-1, ICAM, ALIX, the tetraspanins CD81 and CD63, annexin 5, and TSG101) which are ubiquitous proteins in exosomes.

##### SP-IRIS/ExoView Analysis of the Samples Obtained with the Different Protocols

One of our major concerns was the possible contamination of the samples with the milk fat globules during the extraction or the storage. One possibility would be simple contamination; another is that there might be fusion between vesicles, making it difficult to differentiate between them. As sizes, lipid contents, etc. are similar, one way to differentiate the contaminants and then to know which is the method that obtained the purest population is to determine the markers on the surface of the vesicle, as the exosomal markers are well-known and characterized.

**Figure 7:**
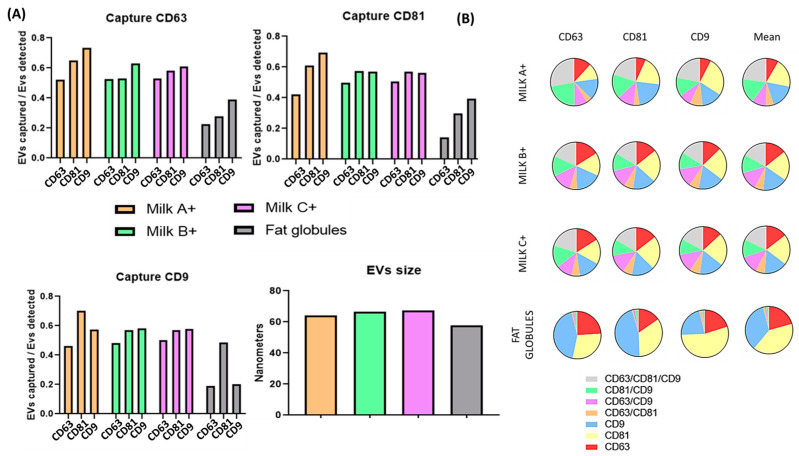
SP-IRIS/ExoView analysis of the vesicles obtained by methods A, B, or C, with the enzymatic treatment and isolated milk fat globules. EV profiles of the exosomes and fat globules highly differ as to the surface markers, as shown on panels (**A**,**B**) of the figure, but not as to the sizes of the vesicles, highlighting the importance of the antibody-based study.

With that objective, we decided to use the ExoView panel of markers. With this technology, we captured with an antibody from an exosomal marker and we labelled other exosomal-specific antibodies. Therefore, the result is highly specific for exosomes, and although other vesicles might share some markers, the whole profile is very specific for each type. There were slight differences between the three methods, as Method A has a lower percentage of CD63 EVs in the capture by the three tertaspanins, as compared with the B and C methods. When vesicles extracted from the different protocols and a sample of purified milk fat globules were compared, we could observe profound differences relative to the markers’ profiles, as can be observed in Figure 7. Fat globules seem to have very little localization of tetraspanins. Although the sizes of the vesicles are all similar (including the fat globules), the markers’ profiles are very different, confirming that the majority of the vesicles obtained from our different protocols were not milk fat globules.

## 4. Discussion

In order to study the therapeutic potential of breast-milk exosomes, it is essential to establish an efficient and robust extraction method that allows them to be obtained in a reproducible way. Defining this protocol is an essential first step for subsequent studies, trials, and commercialization. As mentioned above, there are two components that prevent the obtaining of a clear exosome fraction: fat globules and casein. Currently, existing milk exosome isolation studies have tried to establish protocols in order to eliminate these components, but separately. In this study, we aimed to develop a method that allows the elimination of those two components in the same protocol. We tested three agents to remove casein and three different protocols, in which samples were frozen and centrifuged for human and bovine milk.

### 4.1. Enzymatic Treatment versus Acid Treatment

Our results indicated a marked decrease in protein content after hydrochloric acid treatments, which was indicative of a decrease in the exosome content levels in the samples. This fact was consistent with a few studies that have detected the disappearance of EV-surface-marker proteins such as CD9 and CD81 when using acidic treatments, suggesting that the corrosive nature of the acids could affect the outer structure of the EVs [13].

In general, we observed that human breast-milk showed a decreased coagulation efficiency. This was to be expected, as human breast-milk has a lower percentage of casein (30%, compared to 80% in bovine milk) and less calcium, which is essential for the formation of micelles [16]. Nevertheless, we noticed that casein precipitation in human samples was less efficient than expected, the chymosin derived from Mucor miehei being the only agent capable of catalyzing the precipitation of small amounts of casein in human samples.

In fact, we observed that the enzymatic treatment of exosomes was more suitable than the acidic one. These results were in agreement with other studies that had successfully tested casein rennet precipitation to isolate exosomes in yak milk [17], obtaining an efficient purification and a higher exosome count in rennet-treated samples.

On the other hand, exosome fractions derived from bovine milk by applying a chymosin treatment were visibly cleaner, which could be explained by the fact that these agents are especially selected by the cheese making industry.

Since any extraction procedure, including ultracentrifugation, greatly decreases the concentration of extracted particles, we were not able to observe large decreases in particle values with the different enzymatic treatments (Figure 4B). On the other hand, as indicated, although much purer fractions were obtained, we had to abandon the acid treatment, since the population of exosomes obtained was very poor (Figure 1). The population was reduced at least six-fold, as shown in Figure 1B. We think that enzymatic treatment is the treatment of choice for the isolation of milk exosomes. This is consistent with other studies that have tried the oral administration of bovine milk-derived extracellular vesicles isolated with chymosin.

### 4.2. Pre-Processing Freezing versus Fresh Processing

Another major issue was the presence of fat vesicles that were pelleted with our exosomes. Prior studies had proposed that pre-processing of whole milk prior to storage was required for successful EV isolation [10]. Herein, we explored both the effect of freezing the milk prior to processing, and also the effect of precipitating the casein. Our results showed that Method C (freezing prior to chymosin-incubation and processing) was more effective than Method A or Method B for the efficient purification of milk-derived exosomes. Therefore, we could suggest two things: (I) As Method C is more effective than Method A, the freezing process helps to break down the fat globules, as has been previously described by other authors, and it is an improvement on the method. (II) As Method C gives better results than Method B, this suggests that freezing human breast-milk after the defatting process is more effective than performing the reverse process, possibly due to the fusion of fat globes, or other vesicles, with the exosomal membranes during the freezing process, increasing the risk of obtaining a mixed population.

Purity analysis of the exosomes isolated from human breast-milk with the different protocols showed more marked differences than among those for the exosomes isolated from bovine milk, in which there were no major differences, according to the pre-processing protocol. Nevertheless, some parameters, such as handling procedures, sample preparation, and milk origin, are different among the studies and may have impeded the researchers from establishing robust conclusions. In fact, we observed that pre-treated human milk yielded exosomes free of lipid structures, which suggested that, depending on the species, the degreasing followed by freezing and chymosin treatment could yield pure populations of exosomes.

Although it is well known that the milk fat globule can be easily destroyed by the freezing process (and this could be advantageous when the milk is stored for long periods of time), most of the destroyed globule membranes left were membrane-intercalated particles, whereas a minor portion showed relatively few particles, either in clusters or in apparently random distributions [18]. Therefore, choosing to freeze the milk samples after the extraction might be an advantage, not only for the ease of the process (for the donors and the logistics), but also to eliminate such contaminants as fat globules.

### 4.3. Bovine Milk versus Human Breast-Milk

We aimed to optimize a milk exosome isolation protocol, testing our method in both milk obtained from bovines and human milk. We pursued the finding of the optimal exosome population to use in therapy. On the one hand, cow milk represents a unique source of exosomes, since they can be purified in large amounts and result in a cleaner exosome fraction. On the other hand, we wanted to avoid tolerance and infection-transmission problems associated with using bovine exosomes, but we were unable to obtain an equal purity of human exosomes, as they contained a high amount of casein, which could represent a problem when administrating exosomes as therapeutic agents. However, recent published studies have reported that casein of breast-milk can enhance EV uptake by enabling assembly into functional micelles that can encapsulate other substances [19]. Therefore, further studies should be focused on testing the efficacy of both types of exosomes isolated with our protocol in preclinical trials.

## 5. Conclusions

In conclusion, the three methods analyzed yield similar numbers of vesicles, vesicle sizes, and homologous surface markers. Interestingly, we have developed a method that allows samples to be frozen either prior to shipment for use, or once they have arrived at their destination. This provides a great advantage in the use of milk-derived extracellular vesicles, as it will allow the accumulation of large quantities of excellent-quality vesicles for use in future clinical or therapeutic trials.

We believe that milk EVs are a very interesting source for new therapies, but a robust, reproducible and easy way would be needed to isolate these exosomes while avoiding most contaminants. In this work, we have taken an important step towards being able to use these vesicles in therapeutic approaches, but before regulatory agencies allow their use, we will need to continue working in this direction to be able to obtain a completely pure product.

## Data Availability

Data is unavailable due to privacy restrictions.
